# Does a ketogenic diet as an adjuvant therapy for drug treatment enhance chemotherapy sensitivity and reduce target lesions in patients with locally recurrent or metastatic Her-2-negative breast cancer? Study protocol for a randomized controlled trial

**DOI:** 10.1186/s13063-020-04429-5

**Published:** 2020-06-05

**Authors:** Yan Wang, Ming-Xi Jing, Lei Jiang, Yu-Feng Jia, E. Ying, Hui Cao, Xiang-Yu Guo, Tao Sun

**Affiliations:** 1 Department of Medical Oncology, Cancer Hospital of China Medical University, Liaoning Cancer Hospital & Institute, 44 Xiaoheyan Road, Dadong District, Shenyang, Liaoning Province 110042 P.R. China; 2 Key Laboratory of Liaoning Breast Cancer Research, Cancer Hospital of China Medical University, Liaoning Cancer Hospital & Institute, 44 Xiaoheyan Road, Dadong District, Shenyang, Liaoning Province 110042 P.R. China

**Keywords:** Breast cancer, Ketogenic diet, Irinotecan, Chemotherapy sensitivity, Objective response rate, Randomized controlled trial, Clinical trial

## Abstract

**Background:**

Recent studies have indicated that a ketogenic diet can be used as an adjuvant therapy to enhance sensitivity to chemotherapy and radiotherapy in cancer patients. However, there are no sufficient data and no consistent international treatment guidelines supporting a ketogenic diet as an adjuvant therapy for metastatic breast cancer. Therefore, this trial was designed to observe whether irinotecan with a ketogenic diet can promote sensitivity to chemotherapy and remit target lesions in locally recurrent or metastatic Her-2-negative breast cancer patients.

**Methods/design:**

This trial aims to recruit 518 women with locally recurrent or metastatic breast cancer admitted to the Liaoning Cancer Hospital and Institute (Shenyang, China) in northeast China. All patients will be randomly assigned into the combined intervention group (*n* = 259) or the control group (*n* = 259), followed by treatment with irinotecan + ketogenic diet or irinotecan + normal diet, respectively. The primary endpoints are sensitivity to irinotecan and the objective response rate of target lesions; the secondary endpoints include quality of life scores (EORTC QLQ-C30), progression-free survival, overall survival time, incidence of adverse events, and cost-effectiveness. The endpoints will be evaluated at baseline (before drug administration), during treatment, 4 weeks after treatment completion, and every 3months (beginning 2 months after treatment completion).

**Discussion:**

This trial attempts to investigate whether irinotecan treatment with a ketogenic diet for locally recurrent or metastatic breast cancer among women in northeast China can enhance the disease’s sensitivity to chemotherapy and reduce target lesions.

**Trial registration:**

Chinese Clinical Trial Registry, ID: ChiCTR1900024597. Registered on 18 July 2019. Protocol Version: 1.1, 24 February 2017.

## Background

In China, the treatment procedures for metastatic breast cancer primarily follow the National Comprehensive Cancer Network (NCCN) guidelines, which recommend single-drug treatments for recurrent or metastatic disease [1–3]. Such agents typically include anthracyclines, taxanes, vinorelbine, gemcitabine, capecitabine, and eribulin [3]. However, there are no standard international recommendations for patients with metastatic breast cancer who have developed resistance to anthracyclines and taxanes. Thus, it is difficult to select an effective therapeutic regimen for metastatic breast cancer.

A recent phase II study evaluated the use of NKTR-102 (etirinotecan pegol, a derivative of the topoisomerase I (TOP1) inhibitor irinotecan) among patients with previously treated metastatic breast cancer and revealed a satisfactory therapeutic effect based on an objective response rate (ORR) of 29% [4]. Perez et al. [5] revealed that irinotecan monotherapy (once weekly or every 3 weeks) was safe and effective for patients with breast cancer who developed resistance to anthracyclines and/or taxanes; furthermore, weekly intravenously administered irinotecan administered for four continuous weeks followed by a 2-week rest was more beneficial than intravenously administered irinotecan once every 3 weeks, with slightly fewer adverse events (AEs). Shigeoka et al. [6] retrospectively determined that irinotecan monotherapy (100 mg/m^2^ weekly) was not as effective as salvage chemotherapy in 20 patients with advanced or metastatic breast cancer who were previously treated with doxorubicin or docetaxel. Therefore, further large-sample data are warranted to determine the therapeutic efficacy of irinotecan for advanced breast cancer. These data also indicate that irinotecan monotherapy may be available for patients with metastatic breast cancer who have previously been treated with anthracyclines and/or taxanes.

In recent years, studies have evaluated the role of a ketogenic diet as an adjuvant therapy in enhancing the disease’s sensitivity to chemotherapy and radiotherapy in cancer patients. However, little has been reported on ketogenic diets as an adjuvant therapy for metastatic breast cancer [7]. The combination of a ketogenic diet and high-dose vitamin D3 can decrease Her-2 expression and increase progesterone-receptor expression in breast cancer patients [8]. When ketone bodies function as the main energy source in patients, normal cells in the human body can obtain enough energy to survive; conversely, the growth and metabolism of tumor cells, which are unable to obtain energy from ketone bodies, are inhibited. Although there are limited clinical studies on ketogenic diets for tumor treatment, some animal experiments and case reports have confirmed the effectiveness of a ketogenic diet in the treatment of breast cancer [7–10], which suggests that a ketogenic diet may be a potential strategy for tumor metabolic regulation [11, 12]. Therefore, we hypothesize that the ketogenic diet may be beneficial for breast cancer treatment.

A literature search showed that some clinical protocols targeting a ketogenic diet as an adjuvant treatment for breast cancer have been reported in recent years, such as a ketogenic diet combined with letrozole for treating estrogen-receptor-positive breast cancer (NCT03962647), a ketogenic diet combined with paclitaxel for stage IV breast cancer (NCT03535701), and a ketogenic diet combined with a low-glycemic and insulinemic diet for the rehabilitation of breast cancer patients (Table 1). However, no prospective, randomized controlled trials (RCTs) or articles have assessed irinotecan combined with a ketogenic diet for the treatment of metastatic breast cancer. Therefore, this randomized controlled clinical trial was designed to observe whether irinotecan combined with a ketogenic diet can promote sensitivity to chemotherapy and remit target lesions in locally recurrent or metastatic Her-2-negative breast cancer patients.

**Table 1:** Three clinical trials assessing a ketogenic diet as an adjuvant therapy for breast cancer

Identifiers and status	First posted	Design	Participants	Interventions	Follow-up	Primary outcomes
**NCT03962647; not yet recruiting**	24 May 2019	Single-group assignment, clinical trial	30 patients, ER^+^ breast cancer	Experimental: 2-week ketogenic diet	After 2 weeks of a ketogenic diet	Patients who complete the dietary intervention
**NCT03535701; recruiting**	24 May 2018	Non-randomized, clinical trial	15 patients with stage IV breast cancer (KETO-CARE)	Active comparator:aArm I (standard of care); Experimental: arm II (standard of care, ketogenic diet)	Baseline up to 26 weeks	Adherence and compliance to the ketogenic diet; Changes in psychosocial measures; Changes in physiological outcomes
**NCT02092753; completed**	20 March 2014	Non-randomized, clinical trial	150 patients with breast cancer during the rehabilitation phase	Placebo comparator: standard diet; Experimental: ketogenic diet; Experimental: logi diet	20 weeks spanning 3 phases: 3 weeks of stationary intervention, 16 weeks of outhouse phase, and 1 final week of stationary intervention	Quality of life. This will be assessed by comparing the results of the EORTC QLQ-30 and the QLQ-BR23 questionnaires

The trial will recruit patients in northeast China with locally recurrent or metastatic Her-2-negative breast cancer who have received at least two chemotherapy regimens (such as anthracyclines and taxanes). Irinotecan will be given with a ketogenic diet as an adjuvant therapy. This study is designed to explore whether a ketogenic diet can enhance the sensitivity to irinotecan medication and reduce target lesions with good pharmacoeconomic cost-effectiveness in such patients. If the above clinical benefit requirements can be met, a ketogenic diet may be expected to be a new and effective treatment for such patients. However, if the results are invalid and demonstrate that a ketogenic diet cannot enhance the sensitivity of such patients’ disease to irinotecan medication and has no effect on target-lesion remission, future studies will continue to explore other rational therapeutic opinions for locally recurrent or metastatic Her-2-negative breast cancer.

## Methods/design

### Study design and setting

This prospective, open-label, single-center, parallel RCT will recruit 518 women with locally recurrent or metastatic Her-2-negative breast cancer who have been treated using at least two chemotherapy regimens containing anthracyclines and taxanes. This study aims to assess whether a ketogenic diet combined with irinotecan enhances the sensitivity to chemotherapy and radiotherapy and contributes to target-lesion reduction in such female patients.

The study protocol was approved by the Ethics Committee of Liaoning Cancer Hospital and Institute, China on 1 March 2017 (approval No. 20170234; Additional file 2). All patients will sign an informed consent form (Additional file 3). The study will be performed in line with the Good Clinical Practice (GCP) guidelines and the Declaration of Helsinki formulated by the World Medical Association. The Independent Data and Safety Monitoring Board will be responsible for auditing non-blinded safety and efficacy data from this trial every 3 months. This trial was registered at the Chinese Clinical Trial Registry with approval No. ChiCTR1900024597, Protocol Version: 1.1, 24 February 2017.

**Fig. 1:**
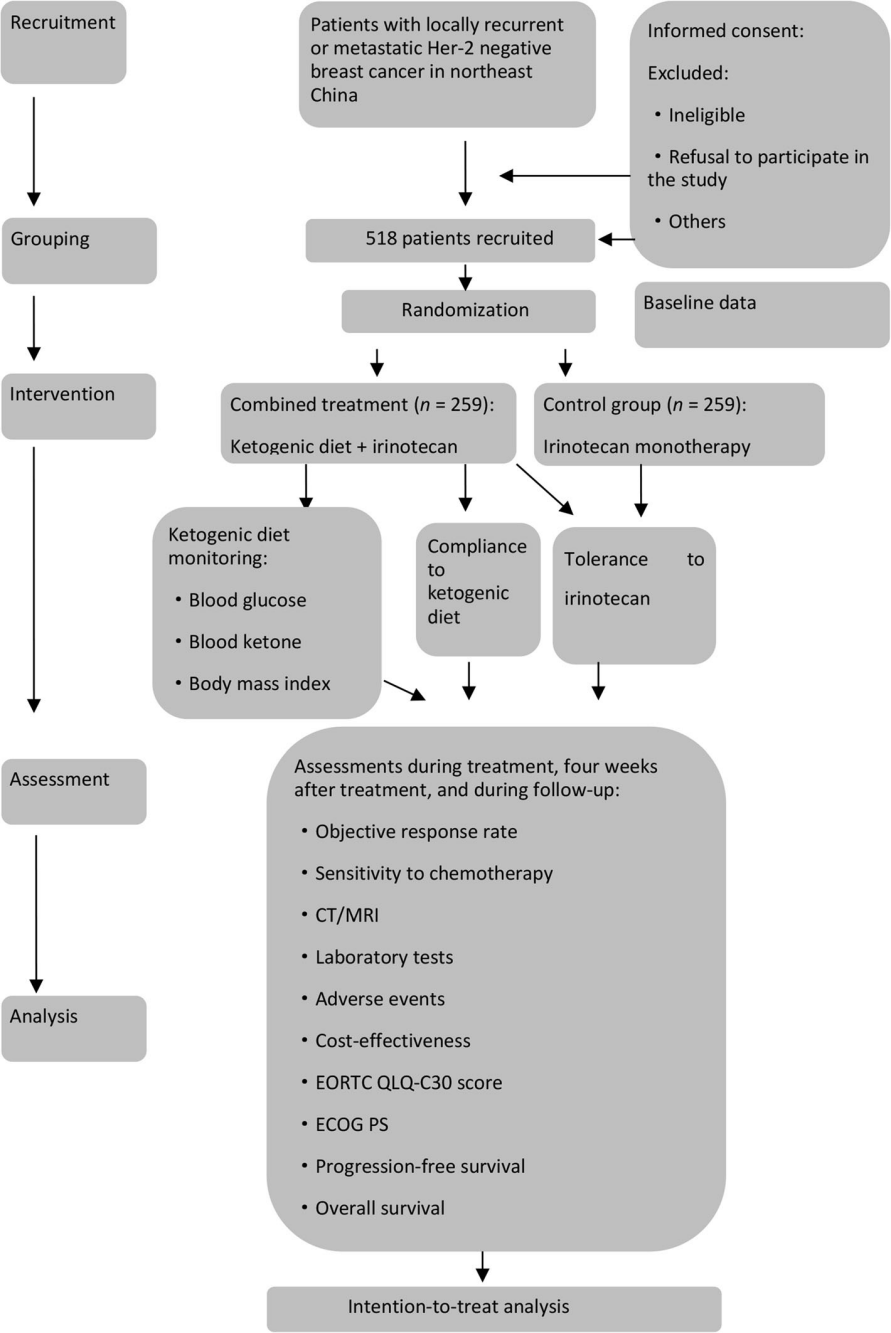
Schedule of enrollment, interventions, and assessments. Laboratory testing will acquire hematological data (hemoglobin, white blood cell count, blood glucose, blood ketones, blood lipids, neutrophil count, and platelet count), biochemical data (total bilirubin, alanine aminotransferase, aspartate aminotransferase, alkaline phosphatase, serum creatinine, total protein, sodium ions, potassium ions, magnesium ions, chloride, calcium, urea, and thyroid function), pregnancy status (if applicable), tumor-marker data (CA153 and carcino-embryonic antigen), routine urinary data (urine glucose, urinary ketones, urine protein), and imaging data (computed tomography/magnetic resonance imaging of target lesions). *EORTC QLQ-C30, version 3* the European Organization for Research and Treatment of Cancer Quality of Life Questionnaire Core-30, *ECOG PS* Eastern Cooperative Oncology Group Performance Status

**Fig. 2:**
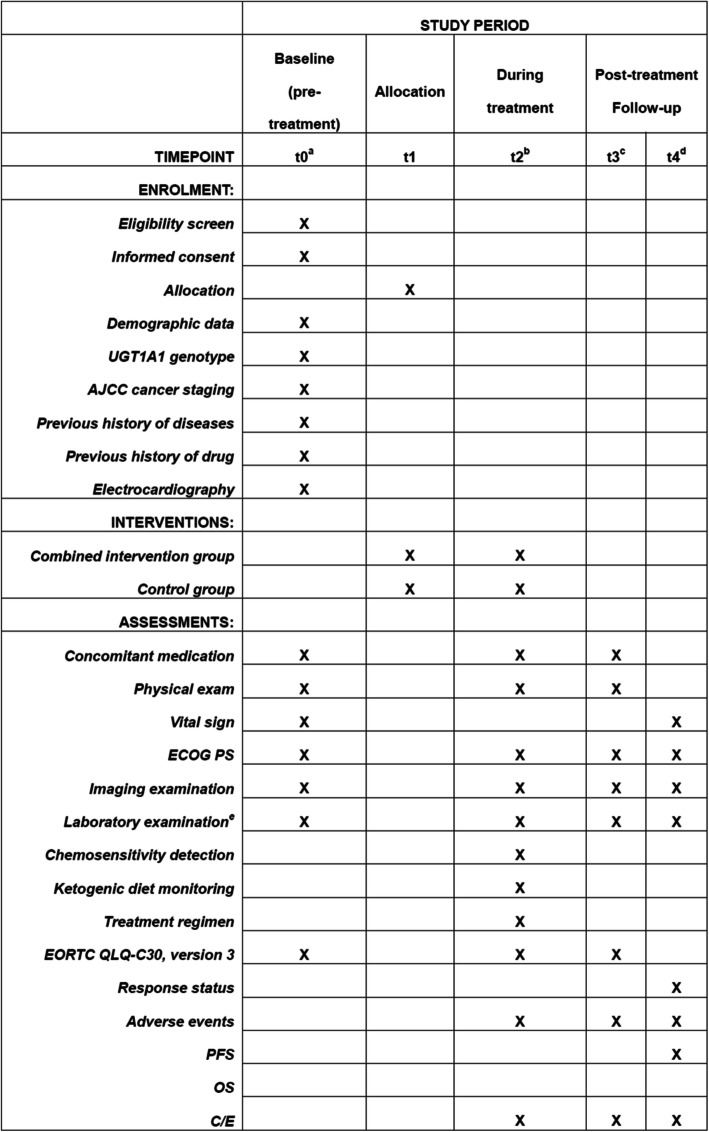
Standard Protocol Items: Recommendations for Interventional Trials (SPIRIT). “a”: baseline evaluations (conducted within 2 weeks of the start of the therapy protocol); “b”: every 6 weeks after inclusion (irinotecan was dosed at 100 mg/m^2^ intravenously on days 1 and 8 of a 3-week cycle); “c”: patients will be monitored for new or existing adverse events for 4 weeks after treatment discontinuation; “d”: follow-up via outpatient appointments and telephone calls will begin after withdrawal of irinotecan and end at patient death. Disease progression information of all patients will be collected and recorded every 3 months beginning 2 months after treatment completion, which will be continued until patient death or the end of PFS follow-up; “e”: laboratory tests will include hematological data (hemoglobin, white blood cell count, blood glucose, blood ketone, neutrophil count, and platelet count), biochemical data (total bilirubin, alanine aminotransferase, aspartate aminotransferase, alkaline phosphatase, creatinine, total protein, sodium ions, potassium ions, magnesium ions, blood chloride, blood calcium, blood urea, and thyroid function index), pregnancy status (if applicable), tumor-marker data (breast-cancer-associated antigen CA153 and carcino-embryonic antigen), and urinary data (urinary sugar, urinary ketones, urinary protein). Tumor measurements during the study will be conducted using computed tomography scans and/or magnetic resonance imaging, with a bone scan if clinically indicated at the time of baseline screening, every 6 weeks after inclusion and 4 weeks after the end of treatment, and then every 3 months thereafter. Treatment response will be evaluated using the Response Evaluation Criteria in Solid Tumors (RECIST version 1.1) criteria. *AJCC* American Joint Committee on Cancer, *ECOG PS* Eastern Cooperative Oncology Group Performance Status Scale, *EORTC QLQ-C30, version 3* the European Organization for Research and Treatment of Cancer Quality of Life Questionnaire Core-30, *PFS* progression-free survival, *OS* overall survival, *C/E* cost/effectiveness ratio

**Fig. 3:**
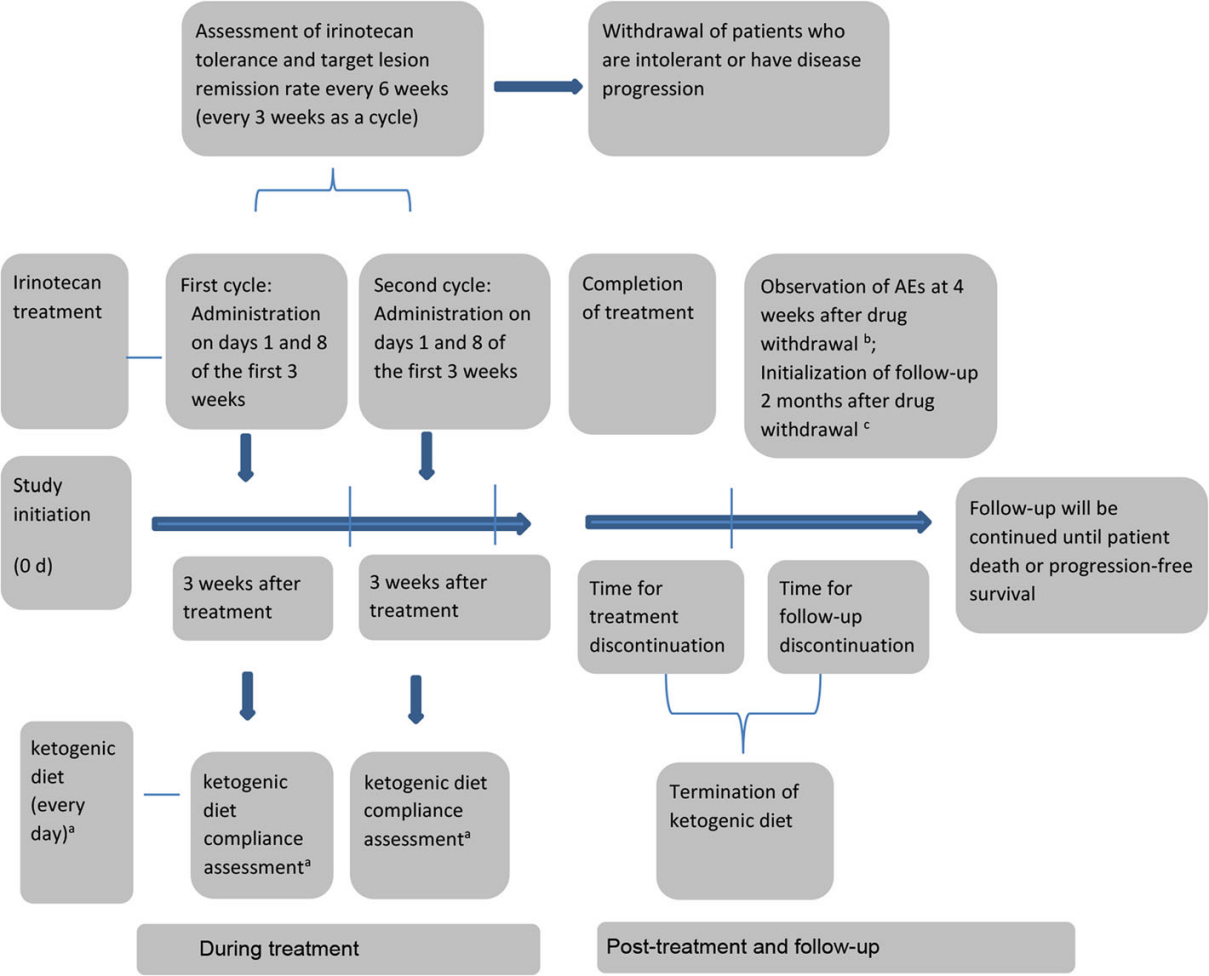
Schedule for assessments of irinotecan and ketogenic-diet therapy. Randomization will be performed 0 days after study initialization. Except for “a” in the figure, the other items indicate the same assessments in the two groups. The details are shown in Fig. 2. “b”: patients will be monitored for new or existing adverse events for 4 weeks after treatment discontinuation. “c”: follow-up for survival will be monitored every 3 months after treatment discontinuation until patient death or study termination

The patients will receive intravenous injections of irinotecan hydrochloride until experiencing disease progression or fulfilling a drug-withdrawal criterion. The trial’s flowchart is shown in Fig. 1. This protocol was written based on the Standard Protocol Items: Recommendations for Interventional Trials (SPIRIT) Checklist (Additional file 1) [13]. The SPIRIT outline for enrollment, interventions, and assessments is shown in Fig. 2. The timeline for the interventions with irinotecan and the ketogenic diet is shown in Fig. 3.

### Eligibility criteria

This trial will recruit 518 female patients with locally recurrent or metastatic Her-2-negative breast cancer who are admitted to the Liaoning Cancer Hospital and and Institute, China, are at least 18 years old, and have received at least two chemotherapy regimens containing anthracyclines and taxanes.

#### Inclusion criteria

- Female patients with histologically or cytologically confirmed locally recurrent or metastatic breast cancer. The Her-2 testing criteria are based on the 2006 American Society of Clinical Oncology (ASCO)/American College of Pathologists (CAP)-approved Her-2 testing guidelines for breast cancer
- Aged 18–70 years
- Body mass index ≥ 24 kg/m^2^, or body fat percentage ≥ 28%
- Tumor tissues with a large amount of tumor cells will be extracted, and TOP1 immunohistochemical testing will be performed in the central laboratory before enrollment, with a TOP1 expression rate ≥ 10%
- Patients with locally recurrent or metastatic breast cancer who are unable to receive radical surgery and have been treated with at least two chemotherapy regimens
  - The regimens must include any anthracycline and any taxane (as adjuvant therapy or/and metastatic therapy) in any combination; if these drugs are contraindicated in the intended patients, they are not required to be used in previous treatments, but they must be recorded in the medical history of the intended patients
  - The disease was not controlled in the latest chemotherapy treatment, i.e., disease progression must have occurred during the last chemotherapy treatment or less than 6 months after the last chemotherapy treatment
  - The use of hormone therapy is allowed in neoadjuvant and/or adjuvant therapy and the treatment of advanced diseases; however, this treatment must be terminated 2 weeks before randomization
- Estimated overall survival ≥ 3 months
- Measurable lesions defined by the Response Evaluation Criteria in Solid Tumors (RECIST) version 1.1 within 2 weeks before enrollment (note: a measurable lesion is confirmed if there has been a previously defined disease progression in the target lesion during radiotherapy) [14]
- Eastern Cooperative Oncology Group (ECOG) Performance Status Scale score 0–2
- Blood routine examinations: white blood cell count ≥ 3.0 × 10^9^/L, absolute neutrophil count ≥ 1.5 × 10^9^/L, and platelet count ≥ 100 × 10^9^/L
- Liver and kidney function tests: total bilirubin ≤ 1.5 times the upper limit of normal (ULN) (a patient with known Gilbert syndrome is eligible if their serum bilirubin level is ≤ 3 × ULN), alanine transaminases ≤ 1.5 times the ULN, aspartate aminotransferase ≤ 1.5 times the ULN, urea nitrogen and serum creatinine ≤ 1.5 times the ULN, and a creatinine removal rate ≥ 50 mL/min (Cockcroft-Gault formula)
- Coagulation function test: international normalized ratio and activated partial thromboplastin time ≤ 1.5 times the ULN
- Intended subjects of childbearing age are negative for serum pregnancy test during screening (within 7 days prior to the first dose) and require effective contraception before enrollment, throughout the study, and 6 months after the last dose
- Provision of informed consent

#### Exclusion criteria

- Patients with homozygous mutations in UGT1A1*6 and/or UGT1A1*28 (such patients are susceptible to irinotecan-induced diarrhea)
- Patients who have received chemotherapy, surgery (such as a major surgery for breast cancer), or molecular-targeted therapy within 4 weeks prior to randomization or who have received hormone therapy or radiotherapy within 2 weeks prior to randomization
- Patients who have participated in other drug clinical trials within 4 weeks prior to randomization
- Uncontrolled hypertension (systolic blood pressure > 150 mmHg and/or diastolic blood pressure > 100 mmHg) or unstable angina; a history of chronic heart failure that meets the New York Heart Association (NYHA) criteria and severe arrhythmias (except for atrial fibrillation and paroxysmal supraventricular tachycardia) that require treatment; a history of myocardial infarction within 6 months prior to randomization
- Patients who have developed central nervous system (CNS) diseases, except for those with asymptomatic CNS metastases who have been treated and met all of the following conditions:
  - Measurable lesions outside the CNS
  - Only supratentorial and cerebellar metastases (i.e., no metastasis to the midbrain, pons, medulla, or spinal cord)
  - CNS diseases not requiring corticosteroid treatment (allowing the use of a stable dose of anticonvulsants)
  - No stereotactic or whole-brain radiotherapy within 2 weeks prior to randomization
  - No evidence of disease progression or bleeding after CNS-targeted therapy; (note: if a new, disease-free CNS metastasis is detected during a screening, the patient must receive radiotherapy and/or surgical treatment for the CNS metastases, and such patients will be excluded from the trial)
- Uncontrollable bone metastasis (i.e., patients who have fractures or have a recent risk of fracture, have a recent schedule for surgery or local radiotherapy, or have other critical conditions)
- Human immunodeficiency virus infection, chronic hepatitis B, hepatitis C, or other infections that have been clinically confirmed at the active phase
- Currently suffering from serious and uncontrollable systemic diseases (e.g., cardiovascular disease, lung disease, or clinically confirmed metabolic disease)
- Metastasis to the liver and kidneys
- Patients undergoing allogeneic organ transplantation who require immunosuppressive therapy
- Known or suspected allergy to the primary agent or any other drug administered during the study
- A history of another primary malignancy, except for bilateral breast cancer, in-situ cervical carcinoma, fully treated non-melanoma skin cancer, and malignant tumors that have been treated and have not relapsed in the past 5 years
- Patients with concurrent metabolic diseases such as carnitine deficiency, carnitine palmitoyl, basal transferase I or II deficiency, carnitine transferase II deficiency, beta-oxidase deficiency, medium-chain acyl-CoA dehydrogenase deficiency, long-chain cyl-CoA dehydrogenase deficiency, short-chain cyl-CoA dehydrogenase deficiency, long-chain 3-hydroxyacyl coenzyme deficiency, medium-chain 3-hydroxyacyl coenzyme deficiency, pyruvate carboxylase deficiency, and porphyria
- Patients with concurrent urinary calculi, history of renal failure or severe renal insufficiency, familial dyslipidemia, severe liver disease, chronic metabolic acidosis, history of pancreatitis, severe diabetes, active gallbladder disease, fat digestive disorder, epilepsy, and severe cardio-cerebrovascular diseases
- Lactating women
- Patients who are unable to cooperate with a ketogenic diet because they have trouble eating

#### Withdrawal criteria

- Failure to recover from treatment-related toxicity to baseline or grade-1 AEs (except for grade-2 hair loss and grade-2 fatigue) within the scheduled 3 weeks (i.e., the inception of each new cycle is over 21 days behind schedule)
- Loss to follow-up
- The patients are randomized to the intervention but do not adhere to the intervention
- Patients for whom the trial medication regimen must be altered
- Presence of concurrent diseases or deterioration of patient’s conditions, or if the investigator believes that the patient cannot continue the study
- Patients with a body mass index ≤ 21 kg/m^2^ are unable to continue the trial
- Patients who have not adhered to the ketogenic diet as prescribed during the treatment, or those who have poor tolerance to the ketogenic diet

## Interventions

### Ketogenic diet

#### Dietary regimen

Patients in the combined intervention group will follow a ketogenic diet during the irinotecan monotherapy. The ketogenic-diet regimen will be developed under the combined guidance of specially trained physicians and dietitians, and nutritional indicators will be regularly monitored. To date, there have been no effective guidelines for a ketogenic diet as an adjuvant therapy for breast cancer in China. Therefore, there are no specific nutritional recipes formulated in the trial, and the intended patients will alter their diet to eat more fatty foods as required by physicians and dietitians. Moreover, the patients are recommended to consume three different meals a day. Diet-specific nutritional counseling will be provided by a registered dietitian during individual face-to-face meetings immediately following the baseline testing visit. The patients will receive weekly telephone calls and/or e-mails from the study dietitian for the remainder of the intervention to review food records and discuss strategies to enhance the patients’ adherence to and enjoyment of their assigned diets. The ketogenic-diet regimen will be self-administered by the patients and their families. The specific-food requirements are as follows:

1. Foods that should be eaten: eggs, leafy greens, above-ground vegetables, high-fat dairy, natural fats, meats, nuts, and seeds
2. Foods that should be forbidden: bread, pasta, rice, potatoes, sugar, honey, and fruits [7, 15]
3. Requirements for fat intake: natural fats should be obtained from foods rich in omega-3, such as salmon, tuna, saury, sardines, etc.; foods containing high levels of monounsaturated fatty acids such as avocado and olive oil [16, 17]

#### Ketogenic-diet monitoring

A follow-up for the ketogenic diet will be performed as follows: the patients will exchange their subjective feelings, including appetite and discomfort, with physician via a 1-h communication every week to adjust the diet guidelines in time. At the initialization of the ketogenic diet, the production of ketone bodies can be detected daily by blood or urinary-ketone tests. For a diet-stable patient, the detection of ketone bodies can be gradually reduced to once or twice a week. According to the patient’s blood-glucose level, the frequency of blood-glucose monitoring is determined based on the patients’ blood-glucose control, and a ratio of blood-glucose to blood-ketone (*G*/*K*) = 1 is defined as an ideal condition, which is used as a measure of ketogenic-diet compliance [18]. Furthermore, the patient’s diet will be adjusted according to the patients’ blood-glucose conditions, and hypoglycemic drugs will be administered if necessary. A complete daily record of ketone levels will be sent to the dietitian for a compliance assessment every 3 weeks, and based on these results, the dietitian will adjust the diet guidelines in a timely manner. The liver and kidney functions, blood lipids, and serum insulin levels will be measured every 3 weeks to assess whether the patient can continue the ketogenic-diet intervention.

#### Assessment of ketogenic-diet compliance

Compliance will be assessed weekly via a telephone call for the patients scheduled to undergo a ketogenic-diet intervention during the trial. The investigators will use a self-made assessment scale for ketogenic-diet compliance that assesses whether the ketogenic-diet guidelines are correct, whether daily food intake can be achieved, whether the patients begin the ketogenic diet at the time instructed, whether the patients independently decide to stop following the ketogenic diet, and whether the patients’ blood-glucose, blood-ketone and urinary-ketone levels, daily diet, and AEs are recorded and reviewed periodically. Each item is scored on a scale from 0 to 3, in which 3 indicates entirely possible, 2 indicates almost possible, 1 indicates nearly impossible, and 0 indicates impossible. Scores of 12–18 indicate complete compliance, 6–11 indicate partial compliance, and 0–5 indicate complete noncompliance. Compliance with the ketogenic diet will be assessed after each irinotecan treatment cycle (every 3 weeks). Patients with complete compliance will continue the trial, whereas those with partial compliance or complete noncompliance will discontinue the ketogenic diet and return to a normal diet.

#### Criteria for ketogenic-diet termination

During the irinotecan monotherapy, patients who develop intolerance to the ketogenic diet, accompanied by severely decreased liver and kidney function and hyperuricemia, will discontinue the ketogenic-diet intervention. Because the ketogenic diet has a certain weight-reducing effect, changes in body mass will be monitored during the intervention, and depending on these changes, the dietitian will decide whether to adjust the diet guidelines. Patients with a body mass index < 22 kg/m^2^ will immediately terminate the ketogenic diet. Discontinuation of the ketogenic diet should last for approximately 2 weeks under the guidance of a nutritionist, followed by a gradual return to a normal diet.

### Drug administration

#### Irinotecan monotherapy

Irinotecan monotherapy (trade name: Aili; batches 180103AG (40 mg) and 171231AG (100 mg), provided by Henrui Medicine, Jiangsu Province, China) will be administered intravenously at an initial dose of 100 mg/m^2^ on days 1 and 8 of each 3-week cycle. The dose will be increased to 125 mg/m^2^ if no AEs occur. The decision to continue treatment or decrease the dose will be based on the incidence and severity of any AEs that arise. All patients will receive irinotecan monotherapy, unless the patients develop drug toxicities, experience cancer progression, or fulfill the withdrawal criteria. Therapeutic efficacy will be evaluated after every 2 treatment cycles [15], and drug tolerance will be assessed under the monitoring of clinical pharmacists in our hospital. Unless they develop toxic reactions, the patients will continue this therapeutic regimen until meeting the criteria for drug withdrawal.

#### Drug-dose adjustment

Dose adjustments and their justifications will be clearly recorded, and all toxicities will be managed using the most appropriate supportive therapy. Treatment will resume at the original dose if medically appropriate after any supportive treatment has provided symptom relief, although a reduced irinotecan dose may be administered if medically necessary. Patients will be withdrawn from the study if the next cycle of treatment is delayed for > 21 days because of AEs.

#### Hematological toxicity

The irinotecan dose in each cycle will be adjusted based on the lowest blood-cell count after the preceding treatment (Table 2). If, after two irinotecan dose reductions, a patient still experiences grade-4 neutropenia or grade-3 granulocyte reduction accompanied by a lack of granulocytes and fever (> 38.5 °C), or a platelet count reduction of grade-3 or greater, the irinotecan dose will not be reduced again. The investigators will discuss whether to continue the treatment on a case-by-case basis in this situation.

**Table 2:** Hematological toxicity criteria for irinotecan dose adjustment

Minimal value		Dose in the next cycle
Neutrophil count (× 10^9^/L)	Platelet count (× 10^9^/L)	
≥ 0.5	… and ≥ 50	No change
< 0.5 or ≥ 0.5 accompanied by fever and lack of granulocytes	… or < 50	Irinotecan dose decreased by 20%

#### Non-hematological toxicity

The irinotecan dose will be reduced by 20% if a patient experiences a non-hematological toxicity other than vomiting and alopecia, such as grade-3 or greater diarrhea and mucositis. Delayed diarrhea will be treated using orally administered loperamide with an initial dose of 4 mg, followed by 2 mg every 2 h until 12 h after the last watery stool. Treatment with loperamide will not exceed 48 h. If necessary, octreotide acetate treatment will also be administered.

#### Liver toxicity

The upcoming treatment cycle will be delayed if bilirubin levels are ≥ 1.5 times the ULN, and treatment will be discontinued if the total bilirubin does not decrease to below this threshold within 3 weeks. Liver protection will be considered in patients with abnormal levels of alanine aminotransferase and/or aspartate aminotransferase and/or alkaline phosphatase in the absence of disease progression. The dose of irinotecan will be adjusted if the transaminase levels do not return to normal within 1 week (Table 3). If liver function subsequently recovers in the next cycle, the irinotecan dose will be increased to the previous level.

**Table 3:** Criteria for irinotecan dose adjustment due to liver toxicity

AST/ALT	Alkaline phosphatase level	Dose adjustment
< 1.5 × ULN	< 5 × ULN	No dose adjustment
1.5 × ULN to < 2.5 × ULN	< 2.5 × ULN	No dose adjustment
2.5 × ULN to < 5 × ULN	< 2.5 × ULN	The irinotecan dose will be reduced by 20%.
> 1.5 × ULN to < 5 × ULN	> 2.5 × ULN to < 5 × ULN	
> 5 × ULN and/or > 5 × ULN (except for bone metastases without any liver damage)		If recovery is not achieved within 3 weeks, the patient will be withdrawn from the trial

*ALT* alanine aminotransferase, *AST* aspartate aminotransferase, *ULN* upper limit of normal

#### Peripheral neurotoxicity

If a patient experiences grade-3–4 peripheral neurotoxicity, which can be life-threatening, the investigators will decide whether to reduce the irinotecan dose by 20% or remove the patient from the trial.

#### Chemotherapeutic drug sensitivity

Solid-tumor tissues or bone-marrow specimens will be extracted from each patient to detect the sensitivity of breast cancer cells to irinotecan using an ATP-TCA kit (Huzhou Haichuang Biotechnology Co., Ltd., China). All testing procedures will be implemented according to the kit’s instructions, and the fluorescence value will be determined.

#### Criteria for re-treatment or treatment delay

Patients will be eligible to receive their next treatment cycle if:

- Their absolute neutrophil count is > 1500/mm^3^
- Their platelet count is > 100,000/mm^3^
- Any treatment-related non-hematological toxicity has resolved to level ≤ 1 or baseline (the exceptions are grade-2 hair loss and grade-2 fatigue)
- Patients who respond well to the medication can continue their therapy with the consent of the study sponsor

#### Medication-cycle delay

Patients will be re-assessed at least once per week. If a patient cannot meet the criteria for re-treatment, planned treatment on day 8 of each cycle will be delayed with a maximum delay of 7 days. Prolonged treatment delay will result in cycle cancellation, and the next cycle will be administered as planned.

#### Drug withdrawal

If a patient gives up the treatment for their own reasons or experiences toxicity that can be life-threatening, the investigators and clinical pharmacist will decide whether to discontinue the medication based on some specific criteria for toxicity response. Time for drug withdrawal and total treatment cycles will be recorded. The patient will be observed for AEs 4 weeks after drug withdrawal and will be followed-up (progression-free survival and overall survival) 2 months after drug withdrawal.

### Outcome measures

The endpoints will be evaluated at baseline (before drug administration), during treatment, 4 weeks after treatment completion, and every 3 months (beginning 2 months after treatment completion).

#### Primary outcome measures

- Sensitivity to irinotecan is determined by various indicators, including the tumor-growth inhibition rate (TGI), chemosensitivity index (CSI), half inhibitory concentration (IC50), and 90% inhibitory concentration (IC90). These indicators are calculated as follows:
  1. TGI = (1 – average fluorescence value in drug treatment group/average fluorescence value in control group) × 100%
  2. CSI-500 indicates the sum of the inhibition rates of the respective drug concentrations. A smaller value indicates a higher inhibition rate of the chemotherapy drug on tumor cells
  3. IC50 indicates a test-drug concentration (TDC) that allows 50% growth inhibition
  4. IC90 indicates a TDC that allows 90% growth inhibition
  5. The sensitivity of chemotherapeutic drugs can be determined according to the TDC, CSI, IC50, and IC90: sensitive, IC50 ≤ 25% TDC; mildly sensitive, IC90 ≤ 100% TDC and IC50 > 25% TDC, or CSI ≤ 300; resistant, IC90 > 100% TDC, IC50 > 25% TDC, and CSI > 300 [19]
- The ORR of the target lesions will be evaluated using the RECIST version 1.1 [14]. The study’s primary endpoint is the ORR, which is defined as the percentage of patients who experience complete or partial cancer shrinkage or disappearance after treatment. The ORR will be calculated by dividing the number of patients who achieve a complete or partial response by the total sample size according the formula:

$$ \mathrm{ORR}=\left[\left(\mathrm{complete}\ \mathrm{response}+\mathrm{partial}\ \mathrm{response}\right)/\mathrm{total}\ \mathrm{cases}\right]\times 100\%. $$

1. Complete response: a complete response will be defined as the disappearance of all target lesions and the reduction of the diameters of all pathological lymph nodes (including target and non-target nodules) to < 10 mm
2. Partial response: a partial response will be defined as a ≥ 30% decrease in the sum of the target-lesions’ greatest diameters relative to the baseline values
3. Progressive disease: progressive disease will be defined as a ≥ 20% increase in the sum of the target-lesions’ greatest diameters relative to the smallest combined value before or during treatment; patients in whom the sum of the tumors’ diameters increases by ≥ 5 mm, or those in whom one or more new lesions appear, will also be considered to be experiencing progressive disease
4. Stable disease: stable disease will be defined as any status that does not meet the definitions of complete response, partial response, or progressive disease, with the minimal sum of the target-lesions’ diameters as a reference

#### Secondary outcome measures

1. The patients’ quality of life will be evaluated using the European Organization for Research and Treatment of Cancer Quality of Life Questionnaire Core 30 (EORTC QLQ-C30), version 3.0 [20]. The scores will be averaged and transformed linearly to obtain a score ranging from 0 to 100, with higher scores indicating a greater response level
2. The progression-free survival will be calculated from the start of randomization to the date of detection of the first instance of tumor progression, treatment failure, or death from any cause. Patients with no available survival data will be censored at the time they are last known to be alive
3. Overall survival time will be calculated from the start of irinotecan treatment to the date of death from any cause. Patients without available survival data will be censored at the time that they are last known to be alive
4. The incidences of grade-3–4 AEs will be evaluated using the National Cancer Institute Common Terminology Criteria (version 4.0) [21]. Irinotecan-related AEs mainly include delayed diarrhea, neutropenia, acute cholinergic syndrome, nausea, and vomiting. Ketogenic diet-related AEs mainly include hypoglycemia, rash, sinus tachycardia, and constipation
5. Cost/effectiveness (*C*/*E*) ratio will be calculated using a pharmacoeconomic cost-effectiveness analysis

#### Other outcome measures

- The patients’ general health will be evaluated using the Eastern Cooperative Oncology Group Performance Status Scale (ECOG PS) [22]
- Laboratory testing will acquire hematological data (hemoglobin, white blood cell count, blood glucose, blood ketones, blood lipids, neutrophil count, and platelet count), biochemical data (total bilirubin, alanine aminotransferase, aspartate aminotransferase, alkaline phosphatase, serum creatinine, total protein, sodium ions, potassium ions, magnesium ions, chloride, calcium, urea, and thyroid function), pregnancy status (if applicable), tumor-marker data (CA153 and carcino-embryonic antigen), routine urinary data (urinary glucose, urinary ketones, urinary protein), and imaging data (computed tomography/magnetic resonance imaging of target lesions). The biological samples collected in this trial will only be used in the present trial and will not be used in other studies

### Sample size estimation

Based on previous evidence and the results of a pilot study [15], this study was designed to test the null hypothesis that the ORR is set at 20% in the combined intervention group and 10% in the control group. Based on a power of 80% (power = 1 − *β*) and a significance level of *α* = 0.05, a sample size of 119 patients is required for each group. Assuming a patient loss rate of 30% due to irinotecan tolerance and ketogenic-diet compliance, we aim to enroll 259 patients in each group.

### Recruitment

- The patients will be mainly recruited from the Department of Medical Oncology, Liaoning Cancer Hospital and Institute, China. This facility focuses on treating advanced breast cancer. This department houses the Liaoning Breast Cancer Clinic, which treats patients from across the entire province
- Recruitment will be performed using leafleting that advertises to advanced breast cancer inpatients and outpatients. After being informed of the trial’s objective and procedures, potentially interested patients or relatives will contact the project manager via their attending physicians, or by telephone, e-mail, or Wechat
- Patients must provide written informed consent if they wish to participate

### Randomization and blinding

The patients will be automatically assigned a random number (1–518) generated using random-number generators with SPSS 22.0 software (IBM, Armonk, NY, USA) and ranked from smallest to largest. As each two participants were selected, one was randomly assigned to the control group and one was randomly assigned to the intervention group. The randomization file will be saved thereafter.

The grouping scheme will be hidden in opaque envelopes: two sets of numbered cards (A refers to the combined intervention group and B refers to the control group) will be placed in envelopes numbered 1–518. The envelopes will be issued to an investigator responsible for the grouping assignments; other investigators will be blinded to the group assignment. This investigator will specify in the envelope who and when to open the randomly assigned envelope. The patients will be treated according to their grouping: irinotecan monotherapy + ketogenic diet for the 259 patients in the combined intervention group and irinotecan monotherapy + ordinary diet for the 259 patients in the control group. No additional analyses (e.g., subgroup and adjusted analyses) are planned.

The data collectors and evaluators will be unaware of the trial protocol. The design is open label with only the data collectors and evaluators being blinded. Thus, unblinding will not occur.

### Data collection and management

This study will use a password-protected electronic data collection system to manage the electronic case report forms (eCRFs). The investigator will complete the eCRFs and upload the data after each patient visit. The investigators will review the eCRFs for accuracy and to ensure that they reflect the patients’ latest results. All data queries will be submitted via the electronic data collection system and answered online by the investigators. The database will be locked after completion of data cleaning.

### Statistical analysis

#### Data description

The analysis will be performed based on the intention-to-treat principle, and no interim analyses will be performed. All statistical analyses will be performed using SPSS 22.0 software (SPSS, IBM, Armonk, NY, USA) and SAS 9.2 software (SAS Institute Inc., Cary, NC, USA). The measurement data will be statistically expressed as the mean, standard deviation, median, minimum and maximum values, and interquartile range. The count data will be statistically described as the number of cases and percentages.

#### Intergroup comparison

A general linear model will be used for the results analysis. For the measurement data, such as the EORTC QLQ-C30 score and the ECOG PS score, the pre-treatment score will be adjusted as a covariate. A completely randomized covariance analysis will be conducted if available. After removing various pre-treatment interference factors, differences in post-treatment scores will be compared, and adjusted means and 95% confidence intervals (CIs) will be calculated. Differences in non-normally distributed data will be compared using the Mann-Whitney *U* test or the paired Wilcoxon rank-sum test. Differences in categorical variables (e.g., the incidence of AEs) will be compared before and after treatment using McNemar’s test. Intergroup indices (overall response rate) will be compared using Pearson’s chi-square test or Fisher’s exact test. Differences in ranked data (e.g., the sensitivity to chemotherapy) will be compared using the Wilcoxon rank-sum test. A correlation analysis between each group of indicators will be performed using Pearson’s correlation analysis (normally distributed data) or Spearman’s correlation analysis (non-normally distributed data).

#### Survival analysis

The Clopper-Pearson method will be used to calculate the response rate and accurate 95% CIs in each group. The progression-free survival, overall survival, and time-to-event endpoint will be analyzed using the Kaplan-Meier method, and the median and corresponding 95% CIs will be determined using the Brookmeyer-Crowley methods. If the proportional hazards assumption holds, a Cox regression model will be used to sort variables that influence the independent risk factors of survival, and calculate hazard ratios and 95% CIs. The *P* value of the Cox model (if the proportional hazards assumption holds) will be derived from the Wald chi-square test. A value of *P* <  0.05 will be considered statistically significant and will not be corrected for multiple comparisons.

#### Economic analysis

An economic cost-effectiveness analysis will be performed.

The following costs will be assessed: (1) medication cost (*C*_*medication*_), which refers to the average direct cost of all drugs during the entire chemotherapy cycle; (2) bed cost (*C*_*bed*_), which refers to the average bed fee during hospitalization; (3) inspection charge (*C*_*inspection*_), which refers to the average cost of the routine blood tests, routine urine tests, liver (kidney) function tests, electrocardiographic examinations, and imaging reviews after completion of the chemotherapy cycle; (4) the cost of treatment (*C*_*treatment*_), which refers to the cost of treatment throughout the entire chemotherapy process; (5) the cost of nursing care (*C*_*nursing*_), which refers to the cost of nursing care throughout the entire chemotherapy period; and (6) the cost of food, which refers to the cost of food throughout the entire chemotherapy period (*C*_*diet*_).

Evaluation indicators include: (1) the ORR for tumor target lesions, which is defined as the percentage of all evaluable cases for patients with complete and partial remission of target lesions; (2) total cost: (*C*_*total*_) = *C*_*medication*_ + *C*_*bed*_ + *C*_*inspection*_ + *C*_*treatment*_ + *C*_*nursing*_ + *C*_*diet*_; and (3) *C*/*E ratio* = *total cost* (*C*_*total*_)/*effect* (E, ORR).

##### Value-added ratio test

Because the cost for irinotecan + ketogenic diet is higher than that for irinotecan monotherapy, we will further calculate the value-added ratio, which is the ratio of how much higher the cost is in the combined intervention group compared with the control group to the increase in therapeutic effectiveness in the combined intervention group compared with the control group.

##### Sensitivity analysis

A sensitivity analysis will be performed based on the principles of pharmacoepidemiology and pharmacoeconomics. The specific method is to assume that the cost of treatment is reduced by 10%, the *C*/*E* ratio is re-calculated, and the *△C*/*△E* of each group is then calculated. Intergroup comparisons will be performed to evaluate the economic benefits.

##### Comparative analysis

Costs will be analyzed using a generalized linear model approach with a gamma distribution to analyze costs using log links to account for possible skewness of the cost data. The ORR serves as the effect analysis indicator. Nonparametric testing will be used for the comparisons of the *C*/*E* and *ΔC*/*ΔE* values in each group. The point estimate of each value and 95% CIs will be calculated.

#### Statistical analysis dataset

The analysis of all demographic data at baseline will be based on a full analysis set, and a safety analysis will be performed on a safety set. The effectiveness of the treatment will be analyzed using the full analysis set and the per-protocol set. For the per-protocol population, the point estimates and 95% CIs for the effectiveness of each treatment group will be calculated. Differences in effectiveness indicators will be calculated to determine the effective rate and clinical benefit. The datasets analyzed during the current study are available from the corresponding author on reasonable request. The principal analyses will be performed in the intention-to-treat population, which includes all patients according to the group to which they are randomly assigned, regardless of the treatment received. The patients who cannot agree to random intervention will not be included in the result analysis. There is no compensation for trial participation. This population is expected to be small because the sample size has been calculated with an expected dropout rate of 30%. Sensitivity analyses will be performed in the per-protocol and as-treated populations, with multiple imputations to account for missing follow-up data [23].

### Safety assessment

#### Adverse events (AEs)

AEs are defined as any adverse medical event that is experienced by a patient regardless of whether it is directly related to treatment. These events indicate any unintentional, unfavorable clinical signs or symptoms (abnormal laboratory data) or any transient drug-related diseases. All AEs will be recorded between the date that the patient signs the informed consent form and that of the termination of treatment. Any potential relationships between the AEs and the study treatment will be considered.

Adverse drug reactions are defined as all toxic and non-intentional responses to any drug dose based on a reasonable possibility of a causal relationship between the dose and AEs.

Serious AEs and serious adverse reactions will be considered present if the patient is at risk of death, requires hospitalization, requires extension of the current hospital stay, develops a significant disability, or causes congenital malformations or birth defects.

Important medical events will be evaluated medically and scientifically to determine whether rapid reporting is necessary. These events may not pose an immediate threat to the patient’s life but may jeopardize the patient’s well-being or may require intervention to prevent serious AEs.

##### Monitoring

To ensure the quality of this RCT, this study will be completed by the Cancer Hospital of China Medical University, Liaoning Cancer Hospital and Institute, China. We will upload data in the Chinese Clinical Trial Registry in a timely manner so that the project management team can collect the data and control errors. The Drug-testing Center of the Cancer Hospital of China Medical University, Liaoning Cancer Hospital and Institute will have the opportunity to obtain the results of the midterm trial and make a decision on it. A qualified clinical trial specialist from China Medical University will be invited to monitor the RCT. The main researchers, YW and TS, are responsible for making protocol decisions, whereas MXJ and XYG are responsible for coordination (e.g., collating/collecting data, performing analyses). TS will be responsible for quality control. YFJ, YE, and HC will be responsible for setting up a quality control committee. The Project Management Group will meet to review trial conduct every 2 months. The Trial Steering Group and Ethics Committee will meet to review conduct throughout the trial period every 3 months. There are no rules for stopping for this trial because the designer of the study protocol believes that it is a safe trial that will not cause any serious adverse reactions to the participants.

##### Recording, assessing, and reporting AEs

All AEs will be recorded on the case report form (CRF), and additional reports will be created for serious AEs. The report for each event will indicate the specific AE in medical terms (vs. self-reported symptoms), the time and date of the AE’s manifestation, and the time and date of its disappearance.

The AEs will be graded using the National Cancer Institute Common Toxicity Criteria (version 4.0) as follows: grade 1 (mild), grade 2 (moderate), grade 3 (severe), grade 4 (life-threatening or disabling), and grade 5 (death) [21].

The potential relationship between each AE and the study drug will be evaluated by the investigators based on the temporal correlation between the AE’s onset and administration of the drug. This relationship will be judged as “irrelevant” (no temporal correlation between the AE and the drug dose, a reasonable causal relationship between the AE and another drug, concomitant disease, or an environmental factor), “unlikely relevant” (the existence of a temporal correlation between the AE and drug dose, but no reasonable causal relationship), “likely relevant” (a reasonable causal relationship between the AE and drug dose, but with a lack of drug withdrawal information), “probably relevant” (a reasonable causal relationship between the AE and drug dose, with drug withdrawal affecting the AE), and “certainly relevant” (a reasonable causal relationship between the AE and drug dose, with AE disappearance after drug withdrawal and re-emergence after re-starting treatment).

The outcome of the AE’s management will be reported as healed with sequelae, healed without sequelae, not healed but no treatment required, not healed but treatment required, and death. If a patient experiences the same AE multiple times, the toxicity grade/severity will be recorded for each instance.

During this study, any serious or medically significant AEs and any abnormal laboratory results will be reported to the Adverse Drug Reaction Monitoring Center, the sponsor, and the study’s Ethics Committee within 24 h via telephone, fax, or mail regardless of any subsequent treatment. After the AEs are verbally reported via telephone, a written report will be delivered via fax. Each report will reflect the investigator’s name, patient’s name, address, telephone number, fax number, and whether the report is considered preliminary or a follow-up. If necessary, the AE report form will be accompanied by the relevant CRF. If a patient dies, the investigators will ensure that the Public Ethics Committees or authorities receive all relevant information.

##### Ethics and informed consent

The study will be initiated only after an independent ethics committee approves the study protocol, informed consent form, recruitment materials, and compensation measures and after the sponsor receives a copy of the Independent Ethics Committee approval with the approval No. research file name (including version number) and approval date. Supplementary treatments and their corresponding informed consent forms will be submitted to the Independent Ethics Committee for prompt review and will only be implemented after they have been approved. On the consent form, the participants will be asked if their data can be used; the participants will be asked if they are unwilling to participate in the trial and choose to withdraw from the trial before the inception of the trial; and the participants will be asked if their data can be used for result analysis if they choose to withdraw from the trial. The participants will also be asked for permission for the research team to share relevant data with people from the hospital who are taking part in the research. This trial involves collecting biological specimens (such as blood samples) for storage.

The study protocol complies with the Good Clinical Practice guidelines, the Declaration of Helsinki, and all applicable local laws and regulations. The study protocol was approved by the Ethics Committee of Liaoning Cancer Hospital and Institute with approval No. 20170234.

##### Protocol modification

All study protocol revisions must be signed, dated, and released by the Department of Medical Oncology, Liaoning Cancer Hospital and Institute, China. The modified study protocol will not be implemented without the approval of the Independent Ethics Committee. No deviations from the study protocol will be permitted; any such deviations will be promptly addressed and corrected by the principal investigator. Any deviations and their reasons must be recorded in the patient’s original medical notes and CRF, which will be preserved by the research centers and study sponsor, respectively.

##### Confidentiality

Confidentiality will be maintained during the periods of data collection and use and will comply with the relevant laws and regulations protecting patient privacy. Patients will provide consent for the collection of their personal data and retain the right to review their personal data and request the modification of incorrect or incomplete data. During the trial, the patients’ personal information will be protected from unauthorized disclosure and from accidental or illegal destruction, loss, or alteration. All sponsor personnel who have access to patient data will ensure that it remains confidential.

##### Compensation

- Patients included in the clinical trial will be monitored and followed-up and may undergo close examinations in our hospital without charge. Each patient will be able to receive transportation and registration fees for admission examination
- The investigator will determine the relevance of any AEs, and claims will be covered for AEs that are considered “relevant” to the study. There is no anticipated harm and compensation for trial participation

## Discussion

To date, the authors have not retrieved any prospective clinical RCTs or clinical research articles on ketogenic-diet-assisted irinotecan for the treatment of metastatic breast cancer. To this end, the authors designed this RCT, with the attempt to observe whether a ketogenic diet combined with irinotecan has a therapeutic efficacy in female patients in northeast China with recurrent or metastatic Her-2-negative breast cancer after treatment with anthracyclines and taxanes. Can this combination therapy affect the chemosensitivity and remission of target lesions? Can such patients experience improved quality of life? What is the economic cost-effectiveness of this combination therapy? Can such patients under this combination therapy ultimately achieve clinical benefits? If the study results are ineffective (the ketogenic diet does not enhance the sensitivity of such patients to irinotecan therapy and has no effect on target-lesion remission), future studies on other treatment options for locally recurrent or metastatic Her-2-negative breast cancer will be continued. If the ketogenic diet can increase the sensitivity to irinotecan monotherapy, and the patients have good compliance with the ketogenic diet, in a future trial, the dietitian will develop specific nutritional ratio rules for the ketogenic diet.

The trial will also have several limitations as follows:

1. Intended patients who will receive irinotecan monotherapy are more likely to be insensitive to chemotherapy and have a low tolerance to standard-dose and long-course chemotherapy regimens due to their poor performance status [24]. Therefore, the collection of outcome indicators may be affected by an insufficient medication course
2. Since the trial is still in its infant stage, it is not possible to estimate and confirm the patients’ compliance with the ketogenic diet and the patients’ tolerance to irinotecan. Therefore, a tolerance assessment will be performed every two treatment cycles of irinotecan (every 6 weeks), and a compliance assessment will be performed every 3weeks to determine whether the patients will continue the follow-up trial; thus, there is a possibility that the protocol may be modified during the trial
3. During the periodic assessments, the trial will be terminated immediately based on the patients’ conditions if over half the patients are not suitable for the ketogenic-diet-assisted chemotherapy intervention or if the target lesions display no obvious reduction after the irinotecan treatment cycle. The patients’ diet will be gradually returned to normal following the advice of the dietitian, and other chemotherapy regimens will be promptly utilized instead of irinotecan to continue treatment

### Trial status

Trial registration: this study was registered with the Chinese Clinical Trial Registry on 18 July 2019 with ID: ChiCTR1900024597); Protocol Version: 1.1, 24 February 2017. Patient recruitment will begin on 2 December 2019 and end on 30 June 2021. The study will end on 31 December 2022. The newest protocol version is Protocol Version 1.1 as of 24 February 2017.

## Supplementary information

**Additional file 1.** Standard Protocol Items: Recommendations for Interventional Trials (SPIRIT) 2013 Checklist: recommended items to address in a clinical trial protocol and related documents.

**Additional file 2.** Ethics approval document.

**Additional file 3.** Informed consent form.

## Data Availability

The datasets generated and/or analyzed during the current study are not publicly available due to the potential for individual and organizational privacy to be compromised. Reasonable requests for parts of the data will be considered by the corresponding author. Data entry according to GCP guidelines was performed by dedicated research nurses and breast-surgery physicians. Data control during the trial is possible. Monitoring will be performed in compliance with GCP guidelines and other rules and regulations to achieve high-quality research and ensure patient safety. At the conclusion of the study, the datasets used and/or analyzed during the current study will be available from the corresponding author on reasonable request. An electronic data collection system will be used for data collection and management in this study. All data will be recorded in an eCRF provided by the sponsor after each patient visit. All original files will be preserved based on the limit set by China’s GCP guidelines, which requires investigators to save clinical trial data for 5 years after termination of the trial. The sponsor maintains ownership of the data, and the investigators will not provide the data in any form to any third party without the sponsor’s written consent. Trial progression, AEs, and data quality will be monitored by an independent data and safety monitoring board, which will be responsible for reporting safety data to the principal investigator. The principal investigator will submit a list of all suspected serious AEs and summary tables to an independent ethics committee every 6 months. TS and YW will have access to the final dataset.

## Abbreviations

NCCN: National Comprehensive Cancer Network
ORR: Objective response rate
ASCO: American Society of Clinical Oncology
CAP: American College of Pathologists
TOP1: Topoisomerase 1
RECIST: Response Evaluation Criteria in Solid Tumors
ECOG: Eastern Cooperative Oncology Group
ULN: Upper limit of normal
NYHA: New York Heart Association
CNS: Central nervous system
AEs: Adverse events
TGI: Tumor-growth inhibition rate
CSI: Chemosensitivity index
IC50: Half inhibitory concentration
IC90: 90% inhibitory concentration
TDC: Test-drug concentration
EORTC QLQ-C30: European Organization for Research and Treatment of Cancer Quality of Life Questionnaire Core 30
ECOG PS: Eastern Cooperative Oncology Group Performance Status Scale
CRF: Case report form
CNY: Chinese Yuan
SPIRIT: Standard Protocol Items: Recommendations for Interventional Trials

## References

[1] Guo W, Hao B, Luo N, Ruan D, Guo X, Chen HJ (2018). Early re-staging and molecular subtype shift surveillance of locally recurrent or metastatic breast cancer: a new PET/CT integrated precise algorithm. Cancer Lett.

[2] Meng XY, Song ST (2018). Diagnosis and treatment of osteoblastic metastasis in patients with breast cancer. Zhonghua Zhong Liu Za Zhi.

[3] Bevers TB, Helvie M, Bonaccio E, Calhoun KE, Daly MB, Farrar WB (2018). Breast cancer screening and diagnosis, Version 3.2018, NCCN Clinical Practice Guidelines in Oncology. J Natl Compr Cancer Netw.

[4] Awada A, Garcia AA, Chan S, Jerusalem GH, Coleman RE, Huizing MT (2013). Two schedules of etirinotecan pegol (NKTR-102) in patients with previously treated metastatic breast cancer: a randomised phase 2 study. Lancet Oncol.

[5] Perez EA, Hillman DW, Mailliard JA, Ingle JN, Ryan JM, Fitch TR (2004). Randomized phase II study of two irinotecan schedules for patients with metastatic breast cancer refractory to an anthracycline, a taxane, or both. J Clin Oncol.

[6] Shigeoka Y, Itoh K, Igarashi T, Ishizawa K, Saeki T, Fujii H (2001). Clinical effect of irinotecan in advanced and metastatic breast cancer patients previously treated with doxorubicin- and docetaxel-containing regimens. Jpn J Clin Oncol.

[7] Klement RJ, Sweeney RA (2016). Impact of a ketogenic diet intervention during radiotherapy on body composition: I. initial clinical experience with six prospectively studied patients. BMC Res Notes.

[8] Branca JJ, Pacini S, Ruggiero M (2015). Effects of pre-surgical vitamin D supplementation and ketogenic diet in a patient with recurrent breast cancer. Anticancer Res.

[9] Morscher RJ, Aminzadeh-Gohari S, Feichtinger RG, Mayr JA, Lang R, Neureiter D (2015). Inhibition of neuroblastoma tumor growth by ketogenic diet and/or calorie restriction in a CD1-Nu mouse model. PLoS One.

[10] Poff AM, Ari C, Seyfried TN, D'Agostino DP (2013). The ketogenic diet and hyperbaric oxygen therapy prolong survival in mice with systemic metastatic cancer. PLoS One.

[11] Smyl C (2016). Ketogenic diet and cancer—a perspective. Recent Results Cancer Res.

[12] Klement RJ, Champ CE, Otto C, Kämmerer U (2016). Anti-tumor effects of ketogenic diets in mice: a meta-analysis. PLoS One.

[13] Chan AW, Tetzlaff JM, Altman DG, Laupacis A, Gøtzsche PC, Krleža-Jerić K (2013). SPIRIT 2013 Statement: defining standard protocol items for clinical trials. Ann Intern Med.

[14] Eisenhauer EA, Therasse P, Bogaerts J, Schwartz LH, Sargent D, Ford R (2009). New response evaluation criteria in solid tumours: revised RECIST guideline (version 1.1). Eur J Cancer.

[15] Crozier JA, Advani PP, LaPlant B, Hobday T, Jaslowski AJ, Moreno-Aspitia A (2016). N0436 (Alliance): a phase II trial of irinotecan with cetuximab in patients with metastatic breast cancer previously exposed to anthracycline and/or taxane-containing therapy. Clin Breast Cancer.

[16] İyikesici MS, Slocum AK, Slocum A, Berkarda FB, Kalamian M, Seyfried TN (2017). Efficacy of metabolically supported chemotherapy combined with ketogenic diet, hyperthermia, and hyperbaric oxygen therapy for stage IV triple-negative breast cancer. Cureus.

[17] Frommelt L, Bielohuby M, Stoehr BJ, Menhofer D, Bidlingmaier M, Kienzle E (2014). Effects of low-carbohydrate, high-fat diets on apparent digestibility of minerals and trace elements in rats. Nutrition.

[18] Jiang B, Zou DJ, Ma XH, Cheng XB, Lu Y, Chen LM (2019). Chinese expert consensus of ketogenic diet intervention for type 2 diabetes [in Chinese]. Shiyong Linchuang Yiyao Zazhi.

[19] Zhao FZ, Xiao SM, Zhao P (2019). Correlation research of in vitro chemosensitivity of chemotherapy drugs in gastric cancer [in Chinese]. Chongqing Yixue.

[20] Zhao H, Kanda K (2004). Testing psychometric properties of the standard Chinese version of the European Organization for Research and Treatment of Cancer Quality of Life Core Questionnaire 30 (EORTC QLQ-C30). J Epidemiol.

[21] Dueck AC, Mendoza TR, Mitchell SA, Reeve BB, Castro KM, Rogak LJ (2015). Validity and reliability of the U.S. National Cancer Institute’s Patient-Reported Outcomes Version of the Common Terminology Criteria for Adverse Events (PRO-CTCAE). JAMA Oncol.

[22] Gradishar WJ, Tjulandin S, Davidson N, Shaw H, Desai N, Bhar P (2005). Phase III trial of nanoparticle albumin-bound paclitaxel compared with polyethylated castor oil-based paclitaxel in women with breast cancer. J Clin Oncol.

[23] Little RJ, D’Agostino R, Cohen ML, Dickersin K, Emerson SS, Farrar JT (2012). The prevention and treatment of missing data in clinical trials. N Engl J Med.

[24] O’Shaughnessy J (2015). Extending survival with chemotherapy in metastatic breast cancer. Oncologist.

