# Supplementary material for: Does a ketogenic diet as an adjuvant therapy for drug treatment enhance chemotherapy sensitivity and reduce target lesions in patients with locally recurrent or metastatic Her-2-negative breast cancer? Study protocol for a randomized controlled trial
Source: Trials. 2020 Jun 5;21:487. doi: 10.1186/s13063-020-04429-5 (PMC7275564; doi:10.1186/s13063-020-04429-5)
Supplement: Supplementary file 2 — Additional file 2. Ethics approval document. [file 13063_2020_4429_MOESM2_ESM.pdf]

The Ethics Committee of the Cancer Hospital of China Medical University Ethical Approval Form

Approval number：20170234

**Project Title**	Does ketogenic diet as an adjuvant therapy for drug treatment enhance chemotherapy sensitivity and reduce target lesions in patients with locally recurrent or metastatic Her-2 negative breast cancer? Study protocol for a randomized controlled trial<br>Version: 1.1 Date: 2017-2-24		
**Affiliation of Application**	Cancer Hospital of China Medical University	**Clinical Trial Affiliation**	Cancer Hospital of China Medical University
**Principal Investigator**	Tao Sun	**Department of Project**	The First Internal Medicine
**Review Date**	2017.3.1	**Review Address**	Medical Ethics Committee of Cancer Hospital of China Medical University
**Review Type**	☐ Meeting Review ☑ Quick Review		
**Documents for review**	Consent form Version 1.1<br>Protocol Version 1.1		
**Review Comments**	The Ethics Committee has carefully reviewed the related content and approved the study to proceed under the current protocol.		
**Other Comments**	None		
**Expiration Date of Document**	2017.3.1-2022.12.28	**Frequency of Review**	☐3 months ☐6 months ☑12 months
**Decision**	Approved<br><br>The Ethics Committee of the Cancer Hospital of China Medical University (Seal)<br>Signature: Haozhe Piao<br><br>Mar. 1, 2017		

Note: The responsibility, staff, effect, procedure and record of the Ethics Committee conforms to ICH-GCP and related regulations of China.

1. **Clinical Investigator:** Tao Sun (Signature) Date: Mar. 1, 2017

辽宁省肿瘤医院医学伦理委员会审查批件

伦理批件编号：20170234

**项目名称及编号**	生酮饮食辅助药物治疗能促进局部复发或转移性 Her-2 阴性乳腺癌患者对化疗的敏感性及靶病灶缓解吗？随机、对照、临床试验方案<br>版本号：1.1 版　　版本日期：2017-2-24		
**申办单位名称**	辽宁省肿瘤医院	**临床试验机构**	辽宁省肿瘤医院
**研究者**	孙涛	**研究所在科室**	内一科
**审查日期**	2017.3.1	**审查地点**	辽宁省肿瘤医院医学伦理委员会
**伦理审查方式**	☐会议审查　　☑快速审查		
**审查文件**	《知情同意》 1.1 版<br>《研究方案》 1.1 版		
**审查意见**	伦理委员会于 2017 年 3 月 1 日对上述内容进行了认真审查。伦理委员会认为该版本的《知情同意》更符合伦理要求，同意变更.		
**其他意见或建议**	无		
**批件有效期**	2017.3.1-2022.12.28	**跟踪审查频率**	☐3 个月　☐6 个月　☑12 个月
**决议**	同意<br>伦理委员会主任/授权者签字：[illegible]<br>2017年 3月 1 日		

注：本伦理委员会的职责、人员组成、作用、操作程序及记录遵循 ICH-GCP 和中国的相关法律法规。

1. 临床研究人员签收：[illegible]　　日期：2017.03.01

地址：辽宁省沈阳市大东区小河沿路 44 号　　邮编：110042　　电话/传真：024-84316632
