# Supplementary material for: Does a ketogenic diet as an adjuvant therapy for drug treatment enhance chemotherapy sensitivity and reduce target lesions in patients with locally recurrent or metastatic Her-2-negative breast cancer? Study protocol for a randomized controlled trial
Source: Trials. 2020 Jun 5;21:487. doi: 10.1186/s13063-020-04429-5 (PMC7275564; doi:10.1186/s13063-020-04429-5)
Supplement: Supplementary file 3 — Additional file 3. Informed consent form. [file 13063_2020_4429_MOESM3_ESM.doc]

**生酮饮食辅助药物治疗能促进局部复发或转移性Her-2阴性乳腺癌患者对化疗的敏感性及靶病灶缓解吗？随机、对照、临床试验方案**

**知情同意书●告知页**

1、研究背景和目的**：**近年研究表明，生酮饮食可作为一种辅助疗法增强肿瘤患者对化疗和放疗的敏感性，但目前有关生酮饮食辅助治疗转移性乳腺癌的临床研究较少，也没有标准的治疗方案，而一些研究显示化疗药物伊立替康对转移性乳腺癌有一定治疗效果。为此，设计此试验方案观察生酮饮食辅助伊立替康药物治疗能否促进局部复发或转移性Her-2阴性乳腺癌患者对化疗的敏感性及靶病灶缓解。

2、研究方法及步骤：本研究是一项前瞻性、单中心、开放性、随机、平行、对照、临床试验。计划纳入来源于中国辽宁省肿瘤医院的518例中国东北地区局部复发或转移性Her-2阴性乳腺癌女性患者，研究的招募对象时间为2019年12月至2021年6月。将518例患者随机分为2组，联合干预组259例采用生酮饮食+伊立替康单药干预，对照组259例采用普通饮食+伊立替康单药干预。两组伊立替康初始给药剂量为100 mg/m2，静脉给药，给药时间点为第1，8天(d1，d8)，每隔3周重复1次。联合干预组在接受相同的伊立替康治疗期间，患者的每日饮食改为生酮饮食。主要观察指标为患者对化疗药物(伊立替康)敏感性的提高和靶病灶客观缓解率(ORR)；次要观察指标是生活质量评分(EORTC QLQ-C30)、无进展生存期(PFS)、总生存时间(OS)、不良事件发生率及成本-效果。主要和次要观察指标评估时间为基线(用药前)、治疗过程中、治疗结束后4周内和治疗结束2个月后每隔3个月1次的随访。

3、研究持续的时间：2019-12至2022-12。

4、受试者风险与受益；

1. 关于研究需要的注射用盐酸伊立替康(商品名：艾力)的药物公司，对试验内容并不知晓，不会影响此临床试验的目的、结果、文章的观点和结果数据的报道。
2. 补偿机制：试验前应明确具体的赔偿标准与办法，由申办方为患者提供“临床试验责任险”。与试验药物有关的不良事件的治疗费用及相应的经济补偿由申办方承担。申请赔偿时，首先需要进行损害归责，通常由研究者判定不良事件与试验药物的关联性，确定为“相关”，即确认是因试验引起的损害，申办者会对其进行相应的理赔。

**二、知情同意书●同意签字页**

本人已仔细阅读“临床研究受试者须知”，已了解这是一项临床研究，临床试验研究者已向我讲解可能存在的不良反应，并对有关问题给予了解答。我在充分了解受试者须知的全部内容以及参加受试带来的利弊后，志愿参加本试验。我已充分理解：

1、作为受试者，我将遵守受试者须知要求，自愿参加本试验，并与研究人员充分合作，如实、客观地向研究人员提供参加本研究前的健康状况及相关情况。

2、本临床试验的结果只用于科研目的，除外国家食品药品监督管理局、本医院伦理委员会、申办单位、研究者或监查员等，我参加试验及试验中的个人资料均属保密，将依照法律规定得到保护。

3、我自愿参加本研究，如果在临床试验中出现不可预知的不良反应，我将得到医生和申办单位妥善积极的免费治疗，如果发生与研究药物有关的严重不良事件，除得到妥善积极的免费治疗外，申办者将会负责由此引起的相关治疗费用及赔偿。

4、我参加本临床试验完全是自愿的，我可以拒绝参加或在任何时间退出试验，而不会遭到歧视或报复，我的医疗待遇与权益亦不会受影响。

联 系 电话： 联 系 电话：

受试者签名： 研究者签名：

日 期： 年 月 日 日 期： 年 月 日

**Does ketogenic diet as an adjuvant therapy for drug treatment enhance chemotherapy sensitivity and reduce target lesions in patients with locally recurrent or metastatic Her-2 negative breast cancer? Study protocol for a randomized controlled trial**

- **Informed consent form for participants**

**PART I: Information sheet**

**Introduction**

Recent studies have indicated that ketogenic diet can be used as an adjuvant therapy to enhance sensitivity to chemotherapy and radiotherapy in cancer patients. However, there are no sufficient data supporting ketogenic diet as an adjuvant therapy for metastatic breast cancer as well as no consistent international treatment guidelines. Irinotecan has been proved to have a certain therapeutic effect on metastatic breast cancer. Therefore, we conceived this trial to observe whether irinotecan with ketogenic diet can promote sensitivity to chemotherapy and remit target lesions in locally recurrent or metastatic breast cancer patients.

**Protocol and Procedures**

This prospective, single-center, open-label, randomized, parallel-controlled trial aims to recruit 518 women with locally recurrent or metastatic breast cancer admitted to Liaoning Cancer Hospital & Institute (Shenyang, China) in Northeast China. All patients will be randomly assigned into combined intervention group (*n* = 259) or control group (*n* = 259), followed by treatment with irinotecan + ketogenic diet or with irinotecan + normal diet, respectively. The primary endpoints are sensitivity to irinotecan and objective response rate of target lesions; secondary endpoints include quality of life scores (EORTC QLQ-C30), progression-free survival, overall survival time incidence of adverse events and cost-effectiveness analysis. The primary and secondary endpoints are evaluated at baseline (before drug administration), during treatment, 4 weeks after treatment completion, and every 3 months (starting at 2 months after treatment completion).

**Duration of the research**

From December 2019 to December 2022.

**Risks and benefits of participants**

1. Irinotecan hydrochloride (trade name: Aili) will be gifted by Jiangsu Hengrui Pharmaceutical Company independent of the trial’s purpose, results and opinions presented in the final report.
2. The sponsor will provide the patient with “clinical drug trial liability insurance”, which will cover the cost of treatment for AEs that are related to the study. The investigator will determine the relevance of any AEs, and claims will be covered for AEs that are considered “relevant” to the study.

**PART II: Certificate of Consent**

I have carefully read the “Notes for Participation in Clinical Trials,” and have understood that this is a clinical study. I have been informed about any adverse reactions that possibly occurring during the trial. I have been given the opportunity to ask questions before I sign, and any questions that I have asked have been answered to my satisfaction. I voluntarily agree to participate as a research subject in this trial after fully understanding the contents of the trial as and the pros and cons if I participate in the trial as follows:

(1) As a research subject, I voluntarily participate in the trial in accordance with the “Notes for Participation in Clinical Trials,” and will fully coordinate with the researcher that I will inform the researcher my health status and related conditions before and after the trial.

(2) The results of this clinical trial are only for scientific research. Except for the State Food and Drug Administration of China, hospital ethics committee, sponsor, researchers or inspectors, unauthorized persons will have no access to my personal data collected in the trial. These personal data will remain confidential and protected by law.

(3) I consent voluntarily to participate in this trial. If unpredictable adverse reactions occur, I will be given timely and free targeted treatments for adverse reactions by doctors and the sponsor. If any serious adverse events related to the study drug occur, besides timely and free targeted treatments, the sponsor will be responsible for relevant treatment costs and compensation.

(4) My participation in this clinical trial is entirely voluntary. I can refuse to participate in or withdraw from the trial at any time without discrimination or retaliation. My medical treatment and benefits will not be affected.

**Tel: Tel:**

**Signature of Participant: Signature of Researcher:**

**Date: (Day/month/year) Date: (Day/month/year)**
